# Quality changes of Huajiao seed oil during refining: A combined approach using traditional methods and multi-omics analysis

**DOI:** 10.1016/j.fochx.2026.104034

**Published:** 2026-05-25

**Authors:** Xiaowei Peng, Bofei Fu, Haibo Liu, Zhuo Chen, Cuilan Fang, Jianquan Kan

**Affiliations:** a College of Food Science, Southwest University, Chongqing 400715, PR China; b Jiulongpo Center for Disease Control and Prevention, Yunhu Road and Panlong Avenue 56, Chongqing 400039, PR China; c College of Food Science and Engineering, Xinyang Agriculture and Forestry University, Xinyang 464000, PR China; d Sichuan Jiaofang Liuxiang Agricultural Technology Limited Company, Nanchong 637548, PR China

**Keywords:** Huajiao seed oil, Refining, Quality changes, Multi-omics analysis

## Abstract

This study systematically investigated the evolution of Huajiao seed oil quality across four refining stages (crude oil, degummed oil, deacidified/deodorized oil, and decolorized oil) using conventional physicochemical analyses integrated with multi-omics approaches. The results demonstrate that refining progressively improved oil quality by significantly reducing the acid value (37.31 to 0.85 mg/g), peroxide value (0.33 to 0.09 g/100 g), glycerophospholipid content (1.76 to 0.11 mg/g), and volatile component content (176.63 to 13.07 μg/g), while enhancing color lightness (7.46 to 70.04). The lipidomic results indicate that the degumming process primarily removed phosphatidylglycerol. Metabolomic analysis revealed that refining resulted in significant loss of fatty acyl glycosides, amino acids, carbonyl compounds, and sesquiterpenoids, whereas fatty alcohols and linoleic acids exhibited better retention. This comprehensive approach elucidated the stage-specific effects of refining on Huajiao seed oil quality and provided a scientific basis for developing precision refining strategies for Huajiao seed oils.

## Highlights

• Refining progressively improves Huajiao seed oil quality by reducing acid value, peroxides, and volatiles.
• Lipidomics reveals degumming primarily removes phosphatidylglycerol among 257 reduced glycerophospholipids.
• Metabolomics showed significant loss of amino acids, carbonyls, and sesquiterpenoids; fatty alcohols retained better.

## 1 Introduction

Huajiao (*Zanthoxylum bungeanum*), a small deciduous tree and perennial plant, belongs to the genus *Zanthoxylum* within the family *Rutaceae*. Native to China, Huajiao has a cultivation history dating back over 2000 years. Today, it has developed into a large-scale industry in provinces such as Sichuan, Shaanxi, Gansu, and Shandong, with enormous annual production (Li et al., 2026). Huajiao seeds, the primary byproduct of Huajiao processing, account for approximately 50% of the dry weight of the fruit (Hou et al., 2019). For a long time, this resource has not been utilized effectively, leading to significant waste. Huajiao seeds are rich in proteins, crude fibers, and minerals; however, their core value lies in their abundant oil content. Research indicates that the oil content of Huajiao seeds can reach 30%. The extracted oil is rich in unsaturated fatty acids, with notable levels of oleic acid, linoleic acid, and α-linolenic acid, making it an important source of essential fatty acids for the human body (Peng, Jiang, et al., 2025; Peng, Zhang, et al., 2025). In addition to fatty acids, Huajiao seed oil contains a wealth of functional bioactive compounds such as natural vitamins, phytosterols, and squalene, which confer significant biological properties, including antioxidant, anti-inflammatory, lipid-lowering, and neuroprotective effects (Zhao et al., 2025).

However, crude Huajiao seed oil obtained by pressing is dark in color and contains various impurities, such as gums, free fatty acids, pigments, volatile off-flavor compounds, and trace metal ions (Liu et al., 2023; Peng et al., 2024). These characteristics render it unsuitable for direct consumption as a commercial edible oil in terms of its flavor, appearance, oxidative stability, and food safety. The presence of these impurities accelerates oxidative rancidity during storage, leading to undesirable flavors and harmful substances, while lowering the smoke point and affecting cooking performance (Chew et al., 2017). Therefore, refining is essential for the purification of crude oil (CO). Traditional oil refining is a systematic physicochemical process involving four core steps: degumming, deacidification, bleaching, and deodorization (Wang et al., 2022). Degumming aims to remove phospholipids and other gum-like impurities; deacidification neutralizes or removes free fatty acids through alkali refining or physical distillation; bleaching employs adsorbents such as activated clay or activated carbon to remove pigments; and deodorization, conducted under high temperature and high vacuum, utilizes steam distillation to eliminate low-molecular-weight volatile compounds responsible for off-flavors (Lv et al., 2024).

The Huajiao seed oil refining process comprises a complex system involving dynamic changes in hundreds to thousands of chemical substances. Traditional quality-monitoring methods, which rely on a limited set of indicators such as acid and peroxide values, are increasingly inadequate to comprehensively reveal the dynamic evolution of intrinsic quality. Multi-omics analysis technologies such as lipidomics, metabolomics, and flavoromics, on the other hand, are more valuable. By systematically analyzing global untargeted changes in lipid molecular species, metabolic small molecules, and volatile flavor compounds throughout the refining stages, a complete quality fingerprint from the raw material to the final product can be constructed. This approach enables the precise identification of critical stages in which active components are lost, and clarifies the pathways through which undesirable substances are formed or eliminated. In general, the combination of the two analytical methods allows for a comprehensive evaluation of oil quality from both macroscopic and microscopic perspectives. This study employs a combination of traditional evaluation methods and omics technologies to investigate the quality of Huajiao seed oil at different refining stages, comprehensively analyzing the changes in basic physicochemical parameters, nutritional characteristics, and trace components of Huajiao seed oil during the refining process. This provides a robust theoretical and data-driven foundation for the development of precise and moderate refining processes.

## 2 Materials and methods

### 2.1 Materials and reagents

CO, degummed oil (DGO), deacidified oil (DAO), and decolorized oil (DCO) of Huajiao seeds were provided by Sichuan Jiaofang Liuxiang Agricultural Technology (Nanchong, China). Analytical-grade potassium dihydrogen phosphate, hydrazine sulfate, potassium hydroxide, zinc oxide, phenylboronic acid, and 2-octanol were purchased from Yuanye Bio-Technology (Shanghai, China). Chromatographic grade α-tocopherol, β-sitosterol, and squalene were purchased from Macklin (Shanghai, China).

### 2.2 Preparation and refining of Huajiao seed oils

#### 2.2.1 Oil preparation

Huajiao seeds were selected, cleaned, dried, and then roasted at 60 °C for 1 h. Subsequently, oil was extracted using a screw press. The pressed oil was filtered to remove the residue and allowed to settle for 24 h. The upper layer was collected to obtain Huajiao seed CO.

#### 2.2.2 Degumming process

Degumming was employed to remove phospholipids from CO. A mixture of hot water (5%, *v*/v) and citric acid (0.3%, v/v) was slowly added to CO. The mixture was stirred continuously at 75 °C for 30 min at a constant speed, after which the stirring rate was reduced to promote the flocculation of the colloidal materials. Following the completion of degumming, the mixture was allowed to stand for 3 h and subsequently centrifuged at 6000 rpm for 20 min at 4 °C. The supernatant was collected to obtain the DGO.

#### 2.2.3 Deacidification and deodorization process

The DGO was transferred into a deacidification apparatus and heated to 240 °C under a reduced pressure of −0.1 MPa. Steam was then introduced, and the oil sample was maintained at this temperature for 120 min to facilitate the removal of free fatty acids and volatile substances. Following this process, heating was terminated and the oil was cooled to 60 °C. Finally, the vacuum was released to yield deacidified (deodorized) oil (DAO).

#### 2.2.4 Decolorization process

The DAO was placed in a reaction vessel and heated to 70 °C. Subsequently, 5% (*w*/*v*) decolorizing agent (comprising a 1:8 mixture of activated carbon and clay) was added. The mixture was maintained at 70 °C with continuous stirring (100 r/min) for 30 min. It was then sequentially filtrated and centrifuged to obtain DCO.

### 2.3 Determination of basic indicators

#### 2.3.1 Glycerophospholipid content

The glycerophospholipid content of the oil samples was determined according to the method described by Li et al. (2023), with minor modifications. Briefly, 3 g of the oil was mixed with zinc oxide (0.5 g). The mixture was subsequently carbonized and ashed. The resulting ash was dissolved in a hydrochloric acid solution. Subsequently, 0.015% (*w*/*v*) hydrazine sulfate and 2.5% (w/v) sodium molybdate solutions were added. The solution was incubated in a boiling water bath for 10 min to induce the colorimetric reaction. The absorbance of the final solution was measured at 650 nm using a microplate reader (Synergy H1; Biotek, USA). Quantification was performed using a calibration curve established using potassium dihydrogen phosphate as the standard. The glycerophospholipid content was calculated using Eq. (1):

(1) $$X=\frac{P}{m}\times \frac{V_{1}}{V_{2}}\times 26.31$$

where *X* is the glycerophospholipid content (mg/g), *P* is the phosphorus content (mg) of the assay solution, *m* is the mass (g) of the oil sample, *V*_1_ is the total dilution volume (mL) of the prepared sample solution, and *V*_2_ is the volume (mL) of the aliquot obtained from *V*_1_.

#### 2.3.2 Acid value, peroxide value, moisture and volatiles, and color

The acid value was determined according to GB 5009.229-2016 (Chinese standard), and the peroxide value was analyzed following GB 5009.227-2023 (Chinese standard). The moisture and volatile matter contents were measured in accordance with GB 5009.236-2016 (Chinese standard). The color (*L*^⁎^, *a*^⁎^, and *b*^⁎^) was measured using a colorimeter (CM-5, Konica Minolta, Tokyo, Japan).

### 2.4 Determination of trace components

#### 2.4.1 α-Tocopherol

The determination of α-tocopherol in oil samples was carried out with reference to the method of Xiang et al. (2023). Briefly, 2 g of oil was dissolved in 6 mL of methanol, then ultrasonically extracted. The supernatant was collected, filtered through an organic filter membrane, and analyzed using high-performance liquid chromatography (1260, Agilent, USA). An α-tocopherol standard was used for qualitative and quantitative analysis simultaneously.

#### 2.4.2 β-sitosterol and squalene

The determination of β-sitosterol and squalene in oil samples was carried out with reference to the method of Peng, Jiang, et al. (2025) and Peng, Zhang, et al. (2025). Briefly, 0.5 g of the oil sample was uniformly mixed with 1 mL of a 1 mol/L KOH–methanol solution, extracted with 5 mL of n-hexane, and washed to pH 7 with a saturated sodium chloride solution. The upper layer was collected, dried over anhydrous sodium sulfate, filtered through an organic filter membrane, and finally analyzed using gas chromatography (GC). β-Sitosterol and squalene standards were used for qualitative and quantitative analysis.

#### 2.4.3 Total polyphenols

The total polyphenol content in the oil samples was determined according to the method described by Jiang et al. (2025). Briefly, an oil sample (0.5 g) was uniformly mixed with 5 mL of methanol. After centrifugation, the supernatant (0.5 mL) was collected, followed by the sequential addition of 0.5 mL of Folin–Ciocalteu reagent and 1 mL of sodium carbonate solution (10%, *w*/*v*). The mixture was allowed to react for 1 h, after which absorbance was measured at 765 nm. Gallic acid was used as a standard for quantitative analysis.

#### 2.4.4 Total flavonoids

The total flavonoid content in the oil samples was determined according to the method described by Zhang et al. (2025). Briefly, an oil sample (0.5 g) was uniformly mixed with 5 mL of methanol. After centrifugation, 2 mL of the supernatant was collected, followed by the sequential addition of 1 mL of sodium nitrite solution (5%, *w*/*v*), 1 mL of aluminum nitrate solution (10%, w/v), and 10 mL of sodium hydroxide solution (5%, w/v). After reacting for 20 min, the absorbance was measured at 510 nm. Rutin was used as a standard for quantitative analysis.

### 2.5 Determination of fatty acid composition

The fatty acid composition of the oil samples was determined as follows: 8 mL of sodium hydroxide–methanol solution (2%) was added to the oil sample (0.5 g) and saponified at 80 °C for 1 h, followed by the addition of 7 mL of boron trifluoride–methanol solution (15%). After cooling, 20 mL of n-heptane and 10 mL of a saturated aqueous sodium chloride solution were added. After layering, 5 mL of the upper layer was collected and dried over anhydrous sodium sulfate, and the supernatant was filtered through an organic membrane for GC analysis (Peng, Jiang, et al., 2025; Peng, Zhang, et al., 2025).

GC analysis: A gas chromatograph equipped with a capillary column of polydi-methylsiloxane strong polar stationary phase (100 m × 0.25 mm, 0.25 μm, Supelco, Bellefonte, PA, USA) and a flame ionization detector (FID) were used to analyze fatty acids. The injection volume was 1.0 μL, detector temperature 280 °C, nitrogen flow rate 1.0 mL/min, and injector temperature 270 °C. The initial column temperature was set to 100 °C, held for 13 min, then increased at a rate of 10 °C/min to 180 °C and held for 6 min, increased at 1 °C/min to 200 °C, held for 20 min, and finally increased at 4 °C/min to 230 °C and held for 10.5 min. A qualitative analysis was performed using a standard mixture of 37 fatty acid methyl esters.

### 2.6 Determination of volatile composition

Volatile compounds in the oil samples were identified according to the method described by Duan et al. (2024). Briefly, 2.0 g of oil and 20 μL of internal standard (0.7646 μg/μL) were transferred into a 20 mL headspace vial and equilibrated at 60 °C for 20 min. Volatile compounds were then extracted by solid-phase microextraction (DVB/CAR/PDMS) for 30 min and analyzed by gas chromatography–mass spectrometry (GC–MS). Quantification was performed using 2-octanol as the internal standard. Two methods were employed to qualify the volatile compositions: calculation of retention indices and searches in the NIST23.L database.

### 2.7 Lipidomics analysis

An 100 μL aliquot of the oil sample was transferred into a centrifuge tube, followed by the addition of 480 μL of extraction solvent (methyl *tert*-butyl ether:methanol = 5:1, *v*/v). The mixture was vortexed for 60 s, ultrasonicated in an ice-water bath for 10 min, and then incubated at −40 °C for 1 h. After centrifugation at 3000 rpm for 15 min at 4 °C, 350 μL of the supernatant was collected into a 2.0 mL Eppendorf tube and dried under vacuum. The dried residue was reconstituted with 500 μL of pre-cooled reconstitution solvent (dichloromethane:methanol = 1:1, v/v, containing isotope-labeled internal standards), vortexed for 30 s, and ultrasonicated in an ice-water bath for 10 min. The sample was then centrifuged at 12,000 rpm for 15 min at 4 °C, and the supernatant was transferred into an autosampler vial for liquid chromatography–mass spectrometry (LC–MS) analysis. Chromatographic separation was performed on a Vanquish ultrahigh performance liquid chromatography system equipped with a Phenomenex Kinetex C18 column (2.1 mm × 100 mm, 2.6 μm). The mobile phase consisted of solvents A (40% water and 60% acetonitrile containing 10 mM ammonium formate) and B (10% acetonitrile and 90% isopropanol containing 10 mM ammonium formate). The injection volume was 2 μL. The MS analysis was performed using an Orbitrap Exploris 120 mass spectrometer controlled by Xcalibur 4.4 software. MS and MS/MS data were acquired in positive and negative ion modes, respectively.

Raw mass spectrometry data were converted to the mzXML format using ProteoWizard. Subsequent data processing, including retention time correction, peak detection, peak extraction, peak integration, and peak alignment, was performed using XCMS software. The parameters were set as follows: minfrac = 0.5 and cutoff = 0.3. Lipids were identified by searching the LipidBLAST 2022 database.

### 2.8 Lipid concomitants analysis

An 100 μL aliquot of each sample was precisely transferred into a 1.5 mL centrifuge tube, followed by the addition of 400 μL of extraction solvent (methanol:acetonitrile = 1:1, *v*/v, containing 2-chloro-*L*-phenylalanine (0.02 mg/mL)). The mixture was vortexed for 30 s and subsequently ultrasonicated for 30 min (5 °C, 40 kHz). The samples were then incubated at −20 °C for 30 min, followed by centrifugation at 13,000*g* for 15 min at 4 °C. The resulting supernatant was collected and evaporated to dryness under a gentle nitrogen stream. The residue was reconstituted in 100 μL of reconstitution solvent (acetonitrile:water = 1:1, *v*/v), vortexed for 30 s, and further extracted by low-temperature ultrasonication for 5 min (5 °C, 40 kHz). After centrifugation at 13,000*g* for 10 min at 4 °C, the supernatant was transferred into an autosampler vial equipped with a microinsert for LC–MS analysis. For quality control (QC) purposes, a pooled QC sample was prepared by mixing 20 μL of the supernatant from each individual sample. LC–MS analysis was performed using an ultrahigh-performance liquid chromatography system coupled with a Q Exactive HF hybrid quadrupole–Orbitrap mass spectrometer (UHPLC-Q Exactive HF). Chromatographic separation was achieved using an ACQUITY UPLC HSS T3 column (100 mm × 2.1 mm, 1.8 μm; Waters, Milford, USA) maintained at 40 °C. The mobile phase consisted of solvent A (95% water and 5% acetonitrile containing 0.1% formic acid) and solvent B (47.5% acetonitrile, 47.5% isopropanol, and 5% water containing 0.1% formic acid). The injection volume was 3 μL. Electrospray ionization in both positive and negative ion modes was used to acquire MS data.

Raw data were processed using Progenesis QI v3.0 (Waters Corporation, Milford, MA, USA) for baseline filtering, peak detection, integration, retention time correction, and alignment, resulting in a data matrix that included retention time, *m*/*z*, and peak intensity. Features were identified by matching the MS and MS/MS spectra against metabolite databases with a mass accuracy threshold of <10 ppm. Lipid concomitants were assigned based on the MS/MS spectral matching scores. The Human Metabolome Database (http://www.hmdb.ca/) and an in-house database from Shanghai Majorbio Bio-Pharm Technology were used.

### 2.9 Statistical analysis

All experiments were conducted in triplicate, and the results presented as the mean ± standard deviation. Data was visualized using an online platform (chiplot.online/; omicshare.com/), and a statistical analysis of differences was conducted using SPSS Statistics 26. Statistical differences were analyzed using one-way analysis of variance, and *p* < 0.05 was defined as statistically significant. The *p*-values were corrected for multiple hypothesis testing on the data using the Holm–Bonferroni method prior to calculation.

## 3 Results and discussion

### 3.1 Basic physicochemical indicators

As shown in Table 1, CO and DGO exhibit relatively high acid values of 37.31 mg/g and 32.51 mg/g, respectively. Following steam distillation deacidification, the acid value dropped markedly to 1.16 mg/g. After subsequent decolorization, it was further reduced to 0.85 mg/g. During high-temperature pressing, oxidation of unsaturated fatty acids in these oils generates hydroperoxides, raising safety concerns (Luo et al., 2024). Refining helps remove these hydroperoxides, thereby lowering the peroxide value. The peroxide value of CO, initially measured as 0.33 g/100 g, which exceeds the edible oil safety limit of 0.25 g/100 g, decreases significantly to 0.03 g/100 g after degumming. Certain phospholipids are amphiphilic and can form micelles or liposomes during hydration. The hydroperoxides generated from oil oxidation are moderately polar and can be encapsulated or adsorbed by these phospholipid micelles, thereby being separated from the oil phase together with the phospholipid aggregates (Nosenko et al., 2024; Zhou et al., 2025). Similar phenomena have also been reported in sunflower seed oil (Nosenko & Zhupanova, 2023). By contrast, the peroxide value of DCO slight increased, likely owing to the elevated temperatures used in the decolorization step (Luo et al., 2024). Although glycerophospholipids possess nutritional benefits (such as aiding lipid regulation and liver protection), excess glycerophospholipids can negatively affect oil quality. A high glycerophospholipid content may cause darkening and cloudiness of the oil and lead to undesirable effects during heating, such as charring, bitter taste, foaming, and smoke emissions (Fine et al., 2016). In the CO group, the glycerophospholipid content was 1.76 mg/g. This is substantially reduced to 0.45 mg/g after hydration degumming and further declines to 0.11 mg/g following decolorization. Furthermore, the moisture and volatile matter content in CO is 1.05 g/100 g, which rises to 4.87 g/100 g in DGO owing to the water added during the degumming process. Subsequent deacidification and decolorization steps significantly lower the content to 0.16 g/100 g and 0.15 g/100 g, respectively.

**Table 1:** Changes in basic physicochemical indicators of Huajiao seed oil at various refining stages.

Indicators	CO	DGO	DAO	DCO
Acid values (mg/g)	37.31 ± 0.37^a^	32.51 ± 0.12^b^	1.16 ± 0.33^c^	0.85 ± 0.13^d^
Peroxide value (g/100 g)	0.33 ± 0.00^a^	0.03 ± 0.01^c^	0.03 ± 0.01^c^	0.09 ± 0.00^b^
Glycerophospholipids (mg/g)	1.76 ± 0.07^a^	0.45 ± 0.04^b^	0.45 ± 0.01^b^	0.11 ± 0.01^c^
Moisture and volatile matter (g/100 g)	1.05 ± 0.14^b^	4.87 ± 0.98^a^	0.16 ± 0.03^c^	0.15 ± 0.02^c^
*L*^⁎^	7.46 ± 0.30^d^	43.69 ± 0.09^b^	27.47 ± 0.13^c^	70.04 ± 0.15^a^
*a*^⁎^	22.01 ± 0.16^c^	28.81 ± 0.02^b^	40.90 ± 0.06^a^	21.75 ± 0.06^c^
*b*^⁎^	12.84 ± 0.49^d^	74.05 ± 0.13^b^	47.36 ± 0.21^c^	95.42 ± 0.01^a^

Note: Different letters (a–d) indicate significant differences (*p* < 0.05) between groups.

**Fig. 1:**
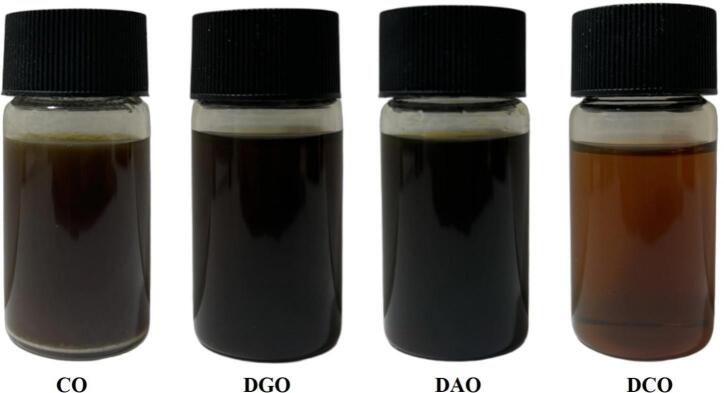
Appearance of Huajiao seed oil at various refining stages.

Color is a crucial sensory parameter of edible oils. As shown in Fig. 1 and Table 1, the color of Huajiao seed oil significantly changed during the refining process. CO exhibited a low (7.46) lightness value (*L*^⁎^) and dark appearance. After refining, the lightness increased notably, with DCO reaching an *L*^⁎^ value of 70.04. Compared with CO, the red–green value (*a*^⁎^) increased for both DGO and DAO. However, after the decolorization step, the *a*^⁎^ value decreases significantly to a level similar to that of CO. By contrast, the yellow–blue value (*b*^⁎^) increased substantially throughout the refining process, rising from 12.84 in CO to 95.42 in DCO, indicating that the final DCO primarily presented a yellow hue.

### 3.2 Trace concomitants

Trace concomitants (lipid-soluble vitamins, phytosterols, and squalene) are vital components of edible oils; however, their levels progressively diminish during refining (Rhazi et al., 2022). As presented in Table 2, the total polyphenol and total flavonoid contents in CO were 2.14 mg/g and 0.28 mg/g, respectively. Following successive refining, these levels declined to 0.65 mg/g and 0.12 mg/g. Notably, deacidification resulted in the most pronounced depletion of total polyphenols, accounting for 62.05% of the total loss. This phenomenon is primarily attributed to the degradation of polyphenolic compounds caused by changes in environmental pH (Zhu et al., 2016). Total flavonoids were also significantly reduced, with losses of 25.93% and 29.41% during deacidification and decolorization, respectively, which are closely related to color changes (Wang et al., 2023). Similarly, the concentrations of α-tocopherol, β-sitosterol, and squalene decreased throughout the refining process. Relative to CO, DCO retained only 30.66% of α-tocopherol, 56.37% of β-sitosterol, and 44.30% of squalene. Importantly, deacidification was responsible for the severe discrete loss of squalene, which accounted for 44.97% of the overall reduction. This is likely attributable to the thermal decomposition of squalene caused by the high temperature.

**Table 2:** Changes in trace concomitants of Huajiao seed oil at various refining stages.

Indicators	CO	DGO	DAO	DCO
α-Tocopherol (μg/g)	259.82 ± 2.36^a^	201.82 ± 13.03^b^	130.28 ± 9.66^c^	79.65 ± 4.35^d^
β-Sitosterol (μg/g)	343.65 ± 2.13^a^	278.80 ± 15.36^b^	225.17 ± 1.95^c^	196.73 ± 6.44^d^
Squalene (μg/g)	169.57 ± 19.46^a^	167.14 ± 5.91^a^	91.98 ± 1.48^b^	75.12 ± 5.99^c^
Total polyphenols (mg/g)	2.14 ± 0.13^a^	1.95 ± 0.14^a^	0.74 ± 0.05^b^	0.65 ± 0.09^b^
Total flavonoids (mg/g)	0.28 ± 0.02^a^	0.24 ± 0.01^b^	0.17 ± 0.03^c^	0.12 ± 0.00^d^

Note: Different letters (a–d) indicate significant differences (*p* < 0.05) between groups.

### 3.3 Fatty acid composition

Fatty acid composition changes of Huajiao seed oil during refining are shown in Table 3. The oleic acid content (36.09–36.43%) remained relatively stable throughout the process, whereas the linoleic acid content marginally increased after degumming (from 25.41% to 26.87%) but decreased after deacidification (from 26.87% to 25.05%). Conversely, palmitoleic acid (from 2.93% to 2.26%) and α-linolenic acid (from 15.84% to 15.02%) contents decreased slightly after the degumming step. A modest increase in the palmitic acid content was observed after deacidification (from 17.37% to 18.84%). In summary, despite some minor fluctuations in individual fatty acids during refining, the overall fatty acid profile of the oil was largely preserved compared with that of the CO.

**Table 3:** Changes in fatty acid composition of Huajiao seed oil at various refining stages.

Fatty acids	Content (%)			
	CO	DGO	DAO	DCO
Palmitic acid	17.35 ± 0.26^a^	17.61 ± 0.02^a^	17.37 ± 0.07^a^	18.84 ± 0.03^b^
Palmitoleic acid	2.93 ± 0.34^a^	2.26 ± 0.38^b^	2.72 ± 0.33^ab^	2.68 ± 0.01^ab^
Stearic acid	2.03 ± 0.33^b^	2.11 ± 0.01^b^	2.68 ± 0.24^a^	1.39 ± 0.13^c^
Oleic acid	36.43 ± 0.62^a^	36.12 ± 0.19^a^	36.26 ± 0.14^a^	36.09 ± 0.46^a^
Linoleic acid	25.41 ± 0.40^a^	26.87 ± 0.62^b^	25.05 ± 0.05^a^	26.09 ± 0.63^ab^
α-Linolenic acid	15.84 ± 0.26^b^	15.02 ± 0.46^a^	14.92 ± 0.12^a^	14.91 ± 0.25^a^

Note: Different letters (a–c) indicate significant differences (*p* < 0.05) between groups.

### 3.4 Volatile composition

**Fig. 2:**
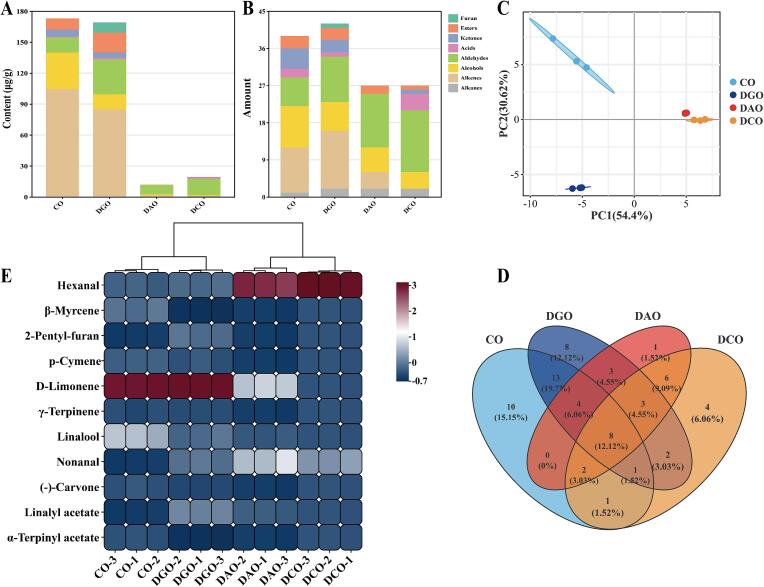
Changes in volatile composition of Huajiao seed oil at various refining stages. (A) Change in content, (B) change in quantity, (C) PCA score plot, (D) heat map of differential volatiles, and (E) Venn diagram.

The volatile profile of Huajiao seed oil significantly changed during the refining process. As shown in Fig. 2A, the maximum total concentration of volatile compounds was 176.63 μg/g. Following treatment with steam distillation deodorization, this concentration decreased significantly to 13.07 μg/g, demonstrating the high efficiency of this deodorization method. Subsequently, a slight increase to 19.25 μg/g was observed in DCO, which might be attributed to the formation of oxidative products under the elevated temperatures of the decolorization step (Sun et al., 2024). Regarding the variety of volatiles (Fig. 2B), DGO exhibited the greatest diversity, with 40 identified compounds, whereas DCO contained the fewest (27 compounds). The refining process substantially influenced both the abundance and spectrum of volatile compounds, with steam deodorization exerting the most pronounced effect. Principal component analysis (PCA) was employed to differentiate the volatile composition and revealed a clear separation among the four oil samples. CO and DGO were distant from the other compounds in the score plot (Fig. 2C), indicating considerable compositional differences. By contrast, DAO and DCO were closely clustered, suggesting a degree of similarity in their volatile profiles. This finding was corroborated by the Venn diagram (Fig. 2D), which highlighted the significant compositional changes throughout the refining process. Only eight volatile compounds were common to all four processing stages, whereas the number of unique compounds in CO, DGO, DAO, and DCO were 10, 8, 1, and 4, respectively. A total of 66 volatile compounds were detected in all samples (Table S1). Among them, 11 were identified as significant markers (VIP > 1, *p* < 0.05) for discriminating the oils (Fig. 2E): hexanal, β-myrcene, 2-pentylfuran, *ρ*-cymene, d-limonene, γ-terpinene, linalool, nonanal, (−)-carvone, linalyl acetate, and α-terpinyl acetate. Notably, hexanal, which is primarily generated by lipid oxidation at high temperatures, is predominantly found in DAO and DCO (Niu et al., 2026). Conversely, d-limonene was abundant in CO and DGO but experienced major loss during the deacidification process. This can be attributed to its volatile nature (Han et al., 2021). Similar findings were reported by Peng, Jiang, et al. (2025) and Peng, Zhang, et al. (2025). In summary, steam distillation effectively reduced the overall volatile content of CO.

### 3.5 Lipidomics

#### 3.5.1 Lipid composition migration

**Fig. 3:**
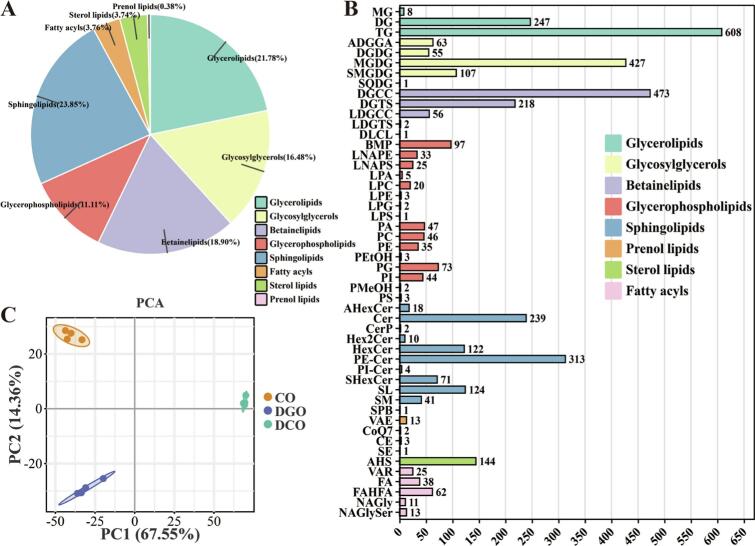
Changes in composition of Huajiao seed oil at various refining stages. (A) Class distribution, (B) subclass distribution, and (C) PCA score plot.

As shown in Fig. 3A, a total of 3962 lipid components were identified in Huajiao seed oil, which were classified into glycerolipids (21.78%), glycosylglycerols (16.48%), betainelipids (18.90%), sphingolipids (23.85%), glycerophospholipids (11.11%), prenol lipids (0.38%), sterol lipids (3.74%), and fatty acyls (3.76%). These lipid components were further divided into 50 subclasses. Among the glycerolipids, triacylglycerols (TGs) and diacylglycerols (DGs) were the most abundant, comprising 608 and 247 species, respectively. Among the glycosylglycerols, monogalactosyldiacylglycerol (MGDG) is the predominant subclass, comprising 427 species. Among beta-lipids, diacylglycerol carboxyhydroxymethylcholine (DGCC) accounted for 473 species. Bis(monoacylglycerol)phosphate (BMP), phosphatidylethanolamine-ceramide (PE-Cer), and acylated hexose sitosterol (AHS) were identified as glycerophospholipids, sphingolipids, and sterol lipids, with 97, 313, and 144 species, respectively (Fig. 3B). These findings indicate that Huajiao seed oil is rich in a diverse range of lipid species, which is consistent with previous research findings (Peng, Jiang, et al., 2025; Peng, Zhang, et al., 2025). After degumming, deacidification (deodorization), and decolorization, the lipid composition of the Huajiao seed oil underwent significant changes. As illustrated in Fig. 3C, PCA revealed substantial Euclidean distances among CO, DGO, and DCO, indicating that the refining process has a marked impact on the lipid profile of Huajiao seed oil.

#### 3.5.2 Glycerophospholipids composition changes

**Fig. 4:**
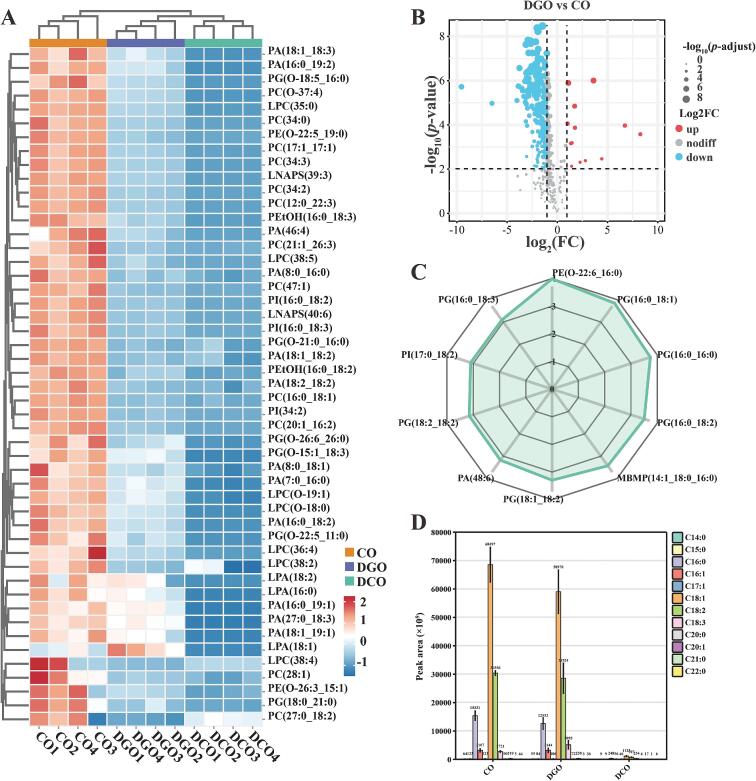
Changes in composition of glycerophospholipids and free fatty acids in Huajiao seed oil at various refining stages. (A) Heat map of glycerophospholipid content, (B) volcano map, (C) radar chart, and (D) changes in free fatty acids.

Degumming is the first step in refining CO, with the core objective of removing the glycerophospholipids in CO, thereby enhancing its stability and improving its appearance and flavor (Yao et al., 2020). Previous studies (Section 3.1) have confirmed that the degumming process can significantly reduce the glycerophospholipid content in CO. However, the specific types of glycerophospholipids removed remain unclear, a question that lipidomic techniques can answer (Aggarwal & Upadhyay, 2026). The results are shown in Fig. 4. In Huajiao seed oil, 440 glycerophospholipids were detected, 49 (*p* < 0.01, VIP > 1) of which were significantly differentially expressed (Fig. 4A). Heat map analysis revealed that the levels of glycerophospholipid molecules, including phosphatidic acid (PA), phosphatidylcholine (PC), phosphatidylinositol (PI), phosphatidylethanolamine (PE), and phosphatidylglycerol (PG), significantly decreased during the refining process, particularly during the degumming stage. To further investigate this, differential analysis of glycerophospholipid molecules in CO and DGO was conducted, and the results are shown in Fig. 4B. In total, the contents of 257 phospholipids were significantly reduced during degumming. Using the log_10_(*p*-value) as an indicator, the top 10 glycerophospholipid molecules with the most significant reduction in content were identified (Fig. 4C). These include PG (16:0_18:1), PG (16:0_16:0), PG (16:0_18:2), PG (18:1_18:2), PG (18:2_18:2), PG (16:0_18:3), PE (O-22:6_16:0), mono-bis(monoacylglycerol) phosphate (MBMP (14:1_18:0_16:0)), PA (48:6), and PI (17:0_18:2). Apparently, PG is a significantly reduced component during the degumming process of Huajiao seed oil, whereas the main losses during the degumming of soybean oil and rapeseed oil are PC, PI, and PE, which may be related to the type of oil (Dos Passos et al., 2022; Zhang et al., 2022). Furthermore, PG is classified as a readily hydratable phospholipid. Its glycerol head group exhibits stronger polarity than the choline group of PC, leading to a greater hydration tendency. During acid degumming, PG remains unaffected by metal ions, thereby achieving higher hydration efficiency (Pan et al., 2024).

Additionally, changes in the free fatty acid (FFA) composition during the refining process of Huajiao seed oil are shown in Fig. 4D. The FFA compositions of CO and DGO were similar, and 12 types of FFAs were detected. The primary FFAs include palmitic acid (C16:0), palmitoleic acid (C16:1), oleic acid (C18:1), linoleic acid (C18:2), and α-linolenic acid (C18:3), which is consistent with the fatty acid composition of Huajiao seed oil reported in previous studies (Peng, Jiang, et al., 2025; Peng, Zhang, et al., 2025). After deacidification (deodorization) and decolorization, the content of each FFA in Huajiao seed oil decreased significantly, which aligns with the results of the acid value determination (Section 3.3).

### 3.6 Changes in lipid concomitant composition

**Fig. 5:**
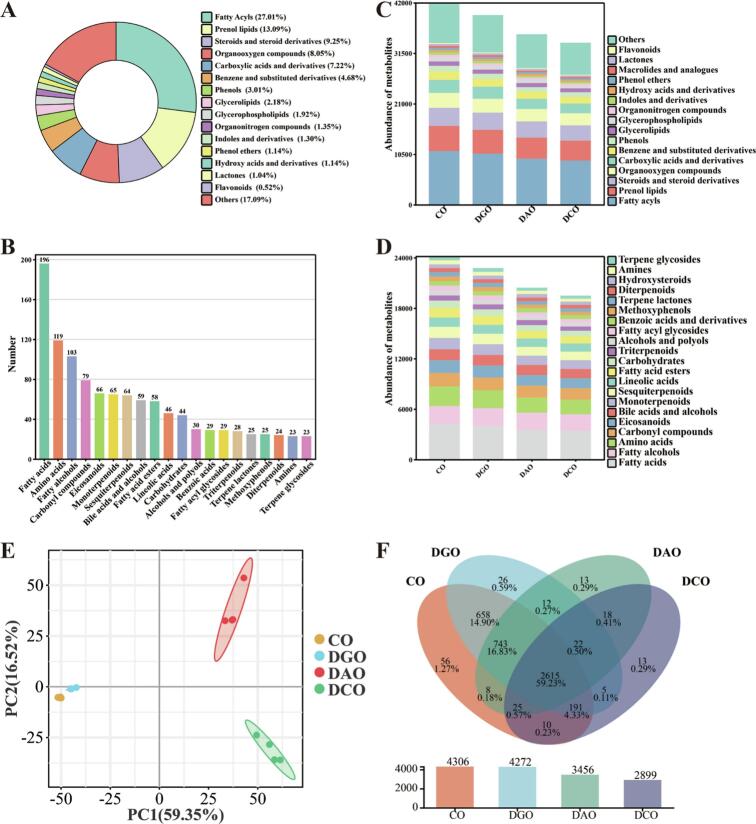
Changes in the composition of lipid concomitants in Huajiao seed oil at various refining stages. (A) Class distribution, (B) subclass distribution, (C) class content, (D) subclass content, (E) PCA score plot, and (F) Venn diagram.

Metabolomics, which enables high-throughput and high-precision profiling of small-molecule lipid concomitants (e.g., fatty acids, phenolics, flavonoids, fat-soluble vitamins, and oxidation products) in oils, offers a novel strategy for elucidating quality changes throughout the refining process (Harkaoui et al., 2025). A total of 4437 lipid concomitants were identified in the four Huajiao seed oil samples. These were broadly categorized into several classes, including fatty acyls (27.01%), prenol lipids (13.09%), steroids and steroid derivatives (9.25%), organooxygen compounds (8.05%), carboxylic acids and derivatives (7.22%), benzene and substituted derivatives (4.68%), phenols (3.01%), and flavonoids (0.52%) (Fig. 5A). At the subclass level, 203 distinct species were detected, predominantly comprising fatty acids (196), amino acids (119), fatty alcohols (103), carbonyl compounds (79), sesquiterpenoids (64), and bile acids and alcohols (59) (Fig. 5B). The levels of these compounds are markedly altered during refining. As illustrated in Fig. 5C, the total content of lipid concomitants was the highest in CO and progressively diminished with subsequent refining steps. Notably, glycerophospholipids, indoles, derivatives, and flavonoids exhibited substantial reductions of 31.67%, 31.01%, and 28.23%, respectively. At the subclass level, pronounced losses were observed in fatty acyl glycosides (26.33%), amino acids (26.33%), carbonyl compounds (27.69%), methoxyphenols (26.07%), and sesquiterpenoids (23.40%) (Fig. 5D). Further analysis revealed that the deacidification (deodorization) stage primarily contributed to a reduction in amino acids, sesquiterpenoids, and methoxyphenols, with losses of 14.85%, 15.60%, and 18.30%, respectively. By contrast, the degumming stage was identified as the principal step responsible for the carbonyl compound loss, which decreased by 9.83% (Sun et al., 2018). Because lipid concomitants are typically considered beneficial to human health, their maximal retention during refining is a key objective (Gonzalez Diaz et al., 2024). The refining process resulted in relatively low losses of several lipid categories, as shown in Fig. 5C: Glycerolipids (11.54%), steroids and steroid derivatives (15.11%), lactones (16.89%), and fatty acids (17.54%). Similarly, at the subclass level, fatty alcohols (10.52%), linoleic acid (12.08%), triterpenoids (14.93%), benzoic acids and derivatives (12.72%), diterpenoids (14.81%), and hydroxysteroids (15.45%) were retained (Fig. 5D). This retention is probably attributable to their resistance to high temperatures and acidic or alkaline environments, and their low hydrophilicity (Lv et al., 2024; Zeb, 2021).

PCA was used to differentiate the lipid concomitants across the distinct refining stages. As illustrated in Fig. 5E, the shorter Euclidean distance between CO and DGO indicates high similarity in their concomitant lipid profiles. By contrast, the longer Euclidean distances between DAO and DGO, as well as between DCO and DAO, suggest that the deacidification and decolorization processes exerted a more pronounced influence on the composition of lipid concomitants in Huajiao seed oil. Venn diagram analysis (Fig. 5F) revealed that 4306, 4272, 3456, and 2899 lipid concomitants were detected in the CO, DGO, DAO, and DCO groups, respectively. Among these, 2615 lipid concomitants were common to all four oil samples. The substantial overlap of the 4207 components between CO and DGO further corroborates the compositional similarity of their lipid concomitants. Furthermore, the numbers of components unique to CO, DGO, DAO, and DCO were 56, 26, 13, and 13, respectively. These findings underscore the progressive reduction in both the total number and unique species of trace components as refining advances, where deacidification and decolorization are the critical stages driving these compositional changes (Lv et al., 2024; Zhang et al., 2026).

To visually delineate the dynamic changes in the lipid concomitants throughout the refining process of Huajiao seed oil, a clustered heat map analysis was performed on the top 50 compounds (*p* < 0.05, VIP > 1); the results are shown in Fig. S1. The contents of several compounds tended to decrease during refining, including azelaic acid, 2,3-dinor prostaglandin E1, 9-oxoOTrE, bolandiol, 13(*S*)-HOTre, FA (18:1 + 3O), and 13-HPOTre. Conversely, the contents of compounds such as 9,12-octadecadiynoic acid, 8-hydroxy-9,10-epoxystearic acid, and dihydroxystearic acid initially increased and then decreased, a pattern potentially linked to the steam distillation process during deacidification (deodorization) (Jeevarathinam et al., 2026). Furthermore, the abundances of certain compounds, including rollitacin, 1-monolinolenin, DG (18:0/18:3), bis(2-ethylhexyl) sebacate, 6-hydroxyoctadec-4-enoic acid, and 9-HODE, increased during the deacidification (deodorization) stage. This increase may be attributed to oxidative decomposition reactions induced by the high temperatures employed in this step (Zhang et al., 2024; Zhu et al., 2025). These observations underscore the stage-specific and compound-dependent effects of the refining process on the lipid concomitant profiles in Huajiao seed oil.

**Fig. 6:**
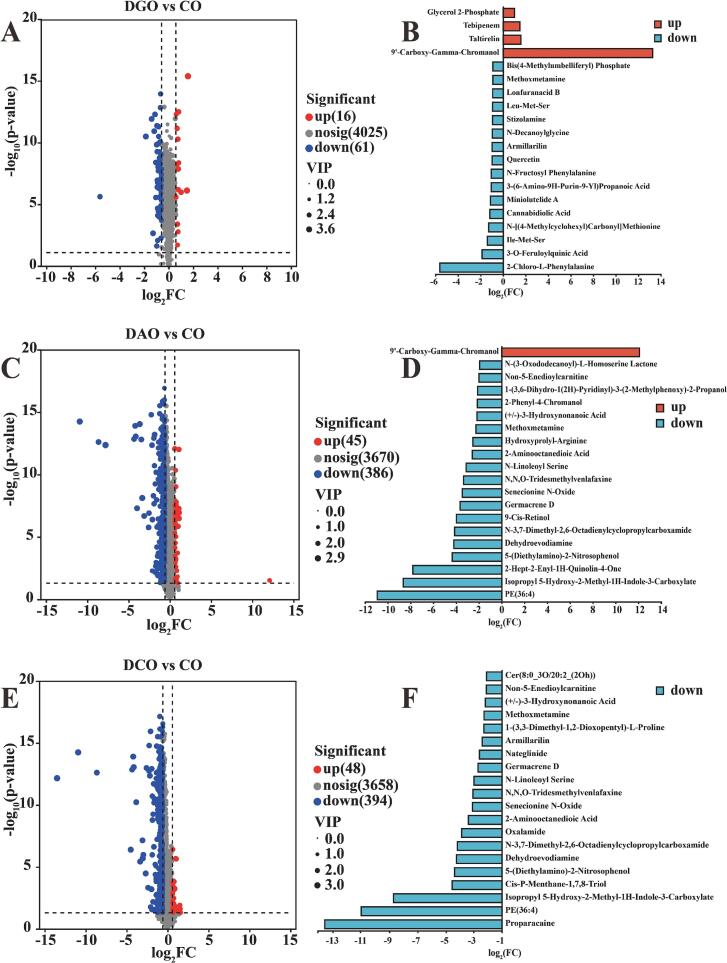
Differences in the composition of lipid concomitants in Huajiao seed oil at various refining stages.

Finally, the impact of the refining process on lipid concomitants was evaluated by comparing changes in their abundance across different stages; the results are presented in Fig. 6. Compared with CO, the abundance of 61 compounds was notably reduced in DGO, primarily 2-chloro-*L*-phenylalanine, 3-O-feruloylquinic acid, Ile-Met-Ser, cannabidiolic acid, and miniolutelide A. In DAO, 386 compounds exhibited decreased levels, predominantly PE (36:4), isopropyl 5-hydroxy-2-methyl-1H-indole-3-carboxylate, 2-hept-2-enyl-1H-quinolin-4-one, dehydroevodiamine, 9-*cis*-retinol, and germacrene D. For DCO, 394 compounds showed reduced levels, primarily proparacaine, PE (36:4), *cis*-*p*-menthane-1,7,8-triol, and 5-(diethylamino)-2-nitrosophenol.

## 4 Conclusion

This study systematically clarified the quality dynamics of Huajiao seed oil throughout the refining process using a combination of traditional analytical methods and multi-omics technologies. The refining process effectively improves oil quality by reducing hazardous components (acid value, peroxide value, and glycerophospholipids) and enhancing sensory properties (color and flavor). Steam deacidification/deodorization proved to be critical for reducing the acid value and eliminating volatile off-flavors. However, refining also causes substantial losses of beneficial trace components, with deacidification identified as the most destructive stage for polyphenols and squalene. Lipidomic analysis revealed that degumming significantly reduced the levels of 257 glycerophospholipids, predominantly phosphatidylglycerols. Metabolomics demonstrated a progressive reduction in lipid concomitants from 4306 to 2899 compounds, with deacidification and decolorization exerting the most profound effects on the metabolomic profile. Flavoromics identified 11 volatile markers that discriminated between the refining stages. Collectively, these findings reveal the tradeoffs between quality improvement and nutrient preservation during refining. This multi-omics characterization provides a comprehensive quality fingerprint and theoretical foundation for developing moderate refining technologies that maximize the removal of undesirable components while minimizing the loss of beneficial bioactive compounds from Huajiao seed oil.

## CRediT authorship contribution statement

**Xiaowei Peng:** Writing – original draft, Methodology, Investigation, Data curation, Conceptualization. **Bofei Fu:** Supervision, Methodology. **Haibo Liu:** Supervision. **Zhuo Chen:** Validation, Supervision, Resources. **Cuilan Fang:** Writing – review & editing. **Jianquan Kan:** Project administration, Methodology, Funding acquisition.

## Declaration of competing interest

The authors declare that they have no known competing financial interests or personal relationships that could have appeared to influence the work reported in this paper.

## Data Availability

Data will be made available on request.
