# Supplementary material for: Quality changes of Huajiao seed oil during refining: A combined approach using traditional methods and multi-omics analysis
Source: Food Chem X. 2026 May 25;36:104034. doi: 10.1016/j.fochx.2026.104034 (PMC13234217; doi:10.1016/j.fochx.2026.104034)
Supplement: Supplementary file 1 — Supplementary material [file mmc1.docx]

**Quality changes of Huajiao seed oil during refining: A combined approach using traditional methods and multi-omics analysis**

**Xiaowei Peng^a,1^, Bofei Fu^a,b,1^, Haibo Liu^a,c^, Zhuo Chen^d^, Cuilan Fang^b,*^, Jianquan Kan^a,*^**

a College of Food Science, Southwest University, Chongqing 400715, PR China

b Jiulongpo Center for Disease Control and Prevention, Yunhu Road and Panlong Avenue 56, Chongqing, 400039, PR China

c College of Food Science and Engineering, Xinyang Agriculture and Forestry University, Xinyang 464000, PR China

d Sichuan Jiaofang Liuxiang Agricultural Technology Limited Company, Nanchong, 637548, PR, China

^1^ Co-first author

* Corresponding Author

E-mail addresses: fangcuilan@163.com (C. Fang); kanjianquan@163.com (J. Kan)

**Table S1**. The changes in the volatile composition of Huajiao seed oil during different refining stages.

| RT (min) | Compounds | RI | Formula | CAS | Content (μg/g) | | | | VIP |
| --- | --- | --- | --- | --- | --- | --- | --- | --- | --- |
|  |  |  |  |  | CO | DGO | DAO | DCO |  |
| 1.40 | Pentane |  | C_5_H_12_ | 109-66-0 |  | 0.09±0.02 | 0.18±0.05 | 0.47±0.12 | 0.61 |
| 1.49 | Acetic acid |  | C_2_H_4_O_2_ | 64-19-7 | 0.80±0.07 |  |  | 0.20±0.05 | 0.56 |
| 1.50 | 2-Methyl-propanal |  | C_4_H_8_O | 78-84-2 |  |  | 0.02±0.00 |  | 0.16 |
| 1.55 | 3-Pentanone |  | C_5_H_10_O | 96-22-0 | 0.01±0.00 |  |  |  | 0.04 |
| 1.60 | Hexane |  | C_6_H_14_ | 110-54-3 |  | 0.06±0.01 | 0.27±0.06 | 0.39±0.11 | 0.37 |
| 1.64 | Ethyl Acetate |  | C_4_H_8_O_2_ | 141-78-6 |  |  | 0.03±0.01 | 0.15±0.06 | 0.42 |
| 1.79 | 2-Butanol |  | C_4_H_6_O | 4170-30-3 | 0.06±0.01 |  | 0.12±0.02 | 0.13±0.04 | 0.18 |
| 1.88 | Propanoic acid |  | C_3_H_6_O_2_ | 79-09-4 | 0.05±0.02 |  |  |  | 0.12 |
| 1.93 | 1-Penten-3-ol |  | C_5_H_10_O | 616-25-1 |  |  | 0.17±0.03 | 0.33±0.05 | 0.43 |
| 1.98 | Pentanal |  | C_5_H_10_O | 110-62-3 | 0.48±0.27 |  | 1.77±0.19 | 2.05±0.38 | 0.71 |
| 2.51 | (E)-2-Pentenal | 729.72 | C_5_H_8_O | 1576-87-0 |  |  | 0.25±0.04 | 0.27±0.05 | 0.25 |
| 2.56 | 1-Pentanol | 734.15 | C_5_H_12_O | 71-41-0 | 1.00±0.54 | 0.34±0.05 | 0.28±0.04 | 0.27±0.05 | 0.39 |
| 3.00 | Hexanal | 774.79 | C_6_H_12_O | 66-25-1 | 7.77±1.83 | 9.25±0.54 | 2.52±0.58 | 8.55±0.14 | 3.00 |
| 4.05 | (E)-2-Hexenal | 832.14 | C_6_H_10_O | 6728-26-3 |  |  | 0.14±0.05 | 0.22±0.01 | 0.30 |
| 4.35 | 1-Hexanol | 844.33 | C_6_H_14_O | 111-27-3 | 0.31±0.17 | 0.08±0.01 |  |  | 0.25 |
| 4.86 | Pentanoic acid | 865.16 | C_5_H_10_O_2_ | 109-52-4 |  |  |  | 0.47±0.14 | 0.87 |
| 4.98 | 2-Heptanone | 869.96 | C_7_H_14_O | 110-43-0 | 0.16±0.02 | 0.26±0.04 |  | 0.27±0.05 | 0.68 |
| 5.31 | Heptanal | 883.80 | C_7_H_14_O | 111-71-7 | 1.75±0.62 | 1.30±0.10 | 1.24±0.48 | 0.90±0.05 | 0.50 |
| 6.35 | α-Thujene | 911.74 | C_10_H_16_ | 2867-05-2 |  | 1.81±0.05 | 0.04±0.01 |  | 0.75 |
| 6.56 | α-Pinene | 915.69 | C_10_H_16_ | 80-56-8 | 1.77±0.32 | 0.73±0.06 |  |  | 0.62 |
| 7.70 | (E)-2-Heptenal | 936.52 | C_7_H_12_O | 18829-55-5 | 0.21±0.03 | 0.34±0.06 | 0.76±0.28 | 0.82±0.05 | 0.35 |
| 8.44 | 1-Heptanol | 950.10 | C_7_H_16_O | 111-70-6 | 1.46±0.72 | 0.64±0.05 | 0.07±0.03 |  | 0.53 |
| 8.62 | β-Phellandrene | 953.44 | C_10_H_16_ | 555-10-2 |  | 2.85±0.11 | 0.04±0.01 |  | 0.93 |
| 9.01 | 1-Octen-3-ol | 960.54 | C_8_H_16_O | 3391-86-4 | 0.37±0.09 | 0.33±0.04 | 0.08±0.02 | 0.07±0.02 | 0.25 |
| 9.40 | 2,3-Octanedione | 967.77 | C_8_H_14_O_2_ | 585-25-1 |  | 0.23±0.02 |  |  | 0.26 |
| 9.76 | β-Myrcene | 974.40 | C_10_H_16_ | 123-35-3 | 11.41±0.82 |  |  |  | 1.78 |
| 9.87 | 2-Pentyl-furan | 976.40 | C_9_H_14_O | 3777-69-3 |  | 9.82±0.87 |  |  | 1.73 |
| 10.20 | (E,E)-2,4-Heptadienal | 982.36 | C_7_H_10_O | 4313-03-5 |  |  | 0.71±0.21 | 0.83±0.18 | 0.46 |
| 11.52 | α-Terpinene | 1005.65 | C_10_H_16_ | 99-86-5 |  | 1.71±0.05 |  |  | 0.72 |
| 12.05 | ρ-Cymene | 1013.98 | C_10_H_14_ | 99-87-6 | 7.64±0.71 | 5.80±1.13 |  |  | 1.28 |
| 12.49 | D-Limonene | 1020.95 | C_10_H_16_ | 5989-27-5 | 79.34±6.51 | 64.02±2.78 | 0.99±0.23 |  | 4.17 |
| 13.30 | 3-Octen-2-one | 1033.63 | C_8_H_14_O | 1669-44-9 | 1.38±0.38 | 1.40±0.29 |  |  | 0.57 |
| 14.09 | β-Ocimene | 1046.01 | C_10_H_16_ | 13877-91-3 |  | 1.68±0.09 |  |  | 0.72 |
| 14.60 | γ-Terpinene | 1054.04 | C_10_H_16_ | 99-85-4 | 3.42±0.88 | 4.55±0.31 |  |  | 1.01 |
| 14.76 | (E)-2-Octenal | 1056.56 | C_8_H_14_O | 2548-87-0 |  | 1.34±0.10 |  |  | 0.64 |
| 16.58 | Heptanoic acid | 1085.11 | C_7_H_14_O_2_ | 111-14-8 |  |  |  | 0.31±0.08 | 0.71 |
| 17.19 | Linalool | 1094.61 | C_10_H_18_O | 78-70-6 | 26.29±3.63 | 9.42±1.69 | 0.21±0.09 |  | 2.40 |
| 17.43 | Nonanal | 1098.47 | C_9_H_18_O | 124-19-6 |  | 10.54±1.23 | 1.01±0.41 | 1.55±0.19 | 1.75 |
| 18.03 | α-Campholenal | 1117.47 | C_10_H_16_O | 4501-58-0 |  | 0.29±0.04 |  |  | 0.30 |
| 18.26 | Neo-alloocimene | 1125.49 | C_10_H_16_ | 7216-56-0 |  | 1.13±0.06 |  |  | 0.59 |
| 19.09 | (E)-2-Nonenal | 1154.26 | C_9_H_16_O | 18829-56-6 | 0.45±0.09 | 1.66±0.18 | 0.15±0.06 | 0.20±0.02 | 0.63 |
| 19.52 | Terpinen-4-ol | 1169.25 | C_10_H_18_O | 562-74-3 | 4.36±0.45 | 3.67±0.42 |  |  | 0.99 |
| 19.88 | α-Terpineol | 1181.70 | C_10_H_18_O | 98-55-5 | 0.81±0.26 |  |  |  | 0.47 |
| 20.26 | Decanal | 1194.83 | C_10_H_20_O | 112-31-2 | 2.58±0.19 | 3.47±0.50 | 0.07±0.02 | 0.07±0.00 | 0.88 |
| 20.46 | (E,E)-2,4-Nonadienal | 1202.75 | C_9_H_14_O | 5910-87-2 |  |  |  | 0.05±0.01 | 0.28 |
| 20.82 | Carveol | 1220.89 | C_10_H_16_O | 99-48-9 | 0.56±0.13 |  |  |  | 0.39 |
| 21.07 | (-)-Carvone | 1233.91 | C_10_H_14_O | 6485-40-1 | 5.06±1.50 | 3.66±0.37 |  |  | 1.03 |
| 21.16 | 4-Oxononanal | 1238.60 | C_9_H_16_O_2_ | 1000314-10-4 |  | 1.83±0.09 |  |  | 0.75 |
| 21.43 | Linalyl acetate | 1252.06 | C_12_H_20_O_2_ | 115-95-7 |  | 13.37±0.24 | 0.24±0.05 |  | 2.03 |
| 21.46 | (E)-2-Decenal | 1253.61 | C_10_H_18_O | 3913-81-3 | 1.47±0.77 | 2.77±0.14 | 0.25±0.04 | 0.18±0.02 | 0.76 |
| 21.62 | 6-Undecanone | 1262.08 | C_11_H_22_O | 927-49-1 | 0.18±0.01 |  |  |  | 0.22 |
| 21.84 | Nonanoic acid | 1273.58 | C_9_H_18_O_2_ | 112-05-0 |  | 1.38±0.27 |  | 0.22±0.09 | 0.66 |
| 21.99 | p-Cymen-7-ol | 1281.07 | C_10_H_14_O | 536-60-7 | 0.11±0.02 |  |  |  | 0.17 |
| 22.03 | (E,Z)-2,4-Decadienal | 1282.94 | C_10_H_16_O | 25152-83-4 |  |  |  | 0.08±0.04 | 0.32 |
| 22.14 | 4-Terpinenyl acetate | 1288.87 | C_12_H_20_O_2_ | 4821-04-9 | 1.78±0.16 | 2.66±0.42 |  |  | 0.77 |
| 22.42 | (E,E)-2,4-Decadienal | 1303.63 | C_10_H_16_O | 25152-84-5 |  | 1.07±0.13 |  | 0.17±0.02 | 0.58 |
| 23.02 | α-Terpinyl acetate | 1336.81 | C_12_H_20_O_2_ | 80-26-2 | 4.68±1.03 |  |  |  | 1.13 |
| 23.26 | (E)-2-Undecenal | 1350.55 | C_11_H_20_O | 53448-07-0 |  |  | 0.11±0.08 | 0.03±0.01 | 0.30 |
| 23.52 | Copaene | 1365.06 | C_15_H_24_ | 3856-25-5 | 0.12±0.03 | 0.09±0.01 |  |  | 0.16 |
| 23.60 | Geranyl acetate | 1369.07 | C_12_H_20_O_2_ | 105-87-3 | 4.33±0.68 | 3.09±0.21 |  |  | 0.96 |
| 24.34 | β-Caryophyllene | 1410.22 | C_15_H_24_ | 87-44-5 | 0.41±0.09 |  |  |  | 0.33 |
| 24.97 | Humulene | 1444.54 | C_15_H_24_ | 6753-98-6 | 0.30±0.09 | 0.23±0.02 |  |  | 0.25 |
| 25.66 | Pentadecane | 1482.78 | C_15_H_32_ | 629-62-9 | 0.04±0.02 |  |  |  | 0.10 |
| 25.80 | α-Muurolene | 1489.91 | C_15_H_24_ | 10208-80-7 | 0.06±0.03 | 0.07±0.02 |  |  | 0.12 |
| 25.92 | β-Bisabolene | 1496.71 | C_15_H_24_ | 495-61-4 | 0.02±0.00 | 0.02±0.00 |  |  | 0.07 |
| 26.25 | (E)-Calamenene | 1514.05 | C_15_H_22_ | 73209-42-4 | 0.04±0.01 | 0.01±0.00 | 0.01±0.01 |  | 0.12 |

Note: RI, Retention index.


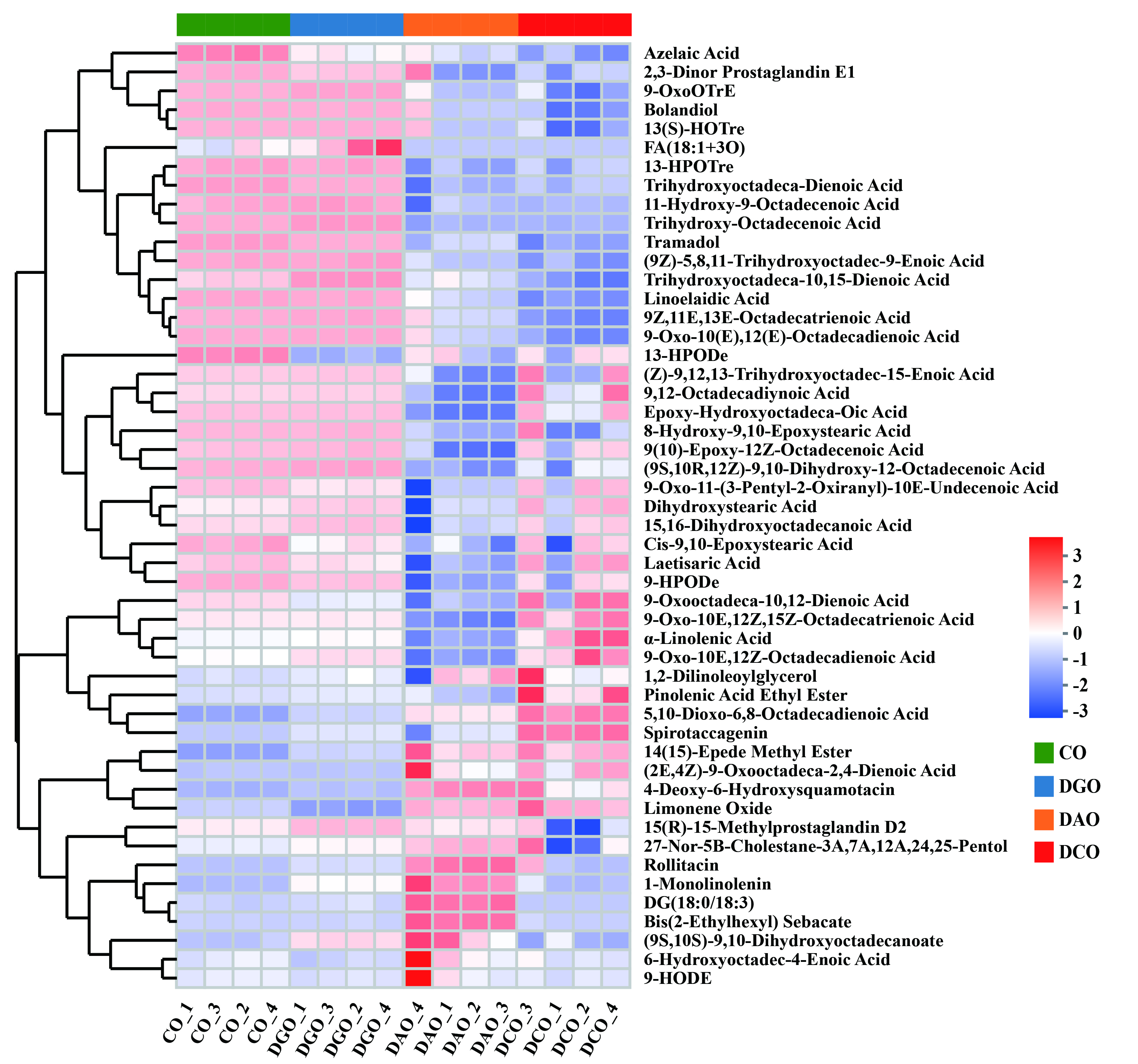


**Fig. S1**. Heatmap analysis of the metabolic composition differences of Huajiao seed oil in different refining stages.
